# Prefrontal cortex, amygdala, and threat processing: implications for PTSD

**DOI:** 10.1038/s41386-021-01155-7

**Published:** 2021-09-20

**Authors:** M. Alexandra Kredlow, Robert J. Fenster, Emma S. Laurent, Kerry J. Ressler, Elizabeth A. Phelps

**Affiliations:** 1 Department of Psychology, Harvard University, Cambridge, MA USA; 2 Division of Depression and Anxiety, McLean Hospital; Department of Psychiatry, Harvard Medical School, Cambridge, MA USA

**Keywords:** Human behaviour, Emotion

## Abstract

Posttraumatic stress disorder can be viewed as a disorder of fear dysregulation. An abundance of research suggests that the prefrontal cortex is central to fear processing—that is, how fears are acquired and strategies to regulate or diminish fear responses. The current review covers foundational research on threat or fear acquisition and extinction in nonhuman animals, healthy humans, and patients with posttraumatic stress disorder, through the lens of the involvement of the prefrontal cortex in these processes. Research harnessing advances in technology to further probe the role of the prefrontal cortex in these processes, such as the use of optogenetics in rodents and brain stimulation in humans, will be highlighted, as well other fear regulation approaches that are relevant to the treatment of posttraumatic stress disorder and involve the prefrontal cortex, namely cognitive regulation and avoidance/active coping. Despite the large body of translational research, many questions remain unanswered and posttraumatic stress disorder remains difficult to treat. We conclude by outlining future research directions related to the role of the prefrontal cortex in fear processing and implications for the treatment of posttraumatic stress disorder.

## Introduction

Post-traumatic stress disorder (PTSD) is a maladaptive and debilitating psychiatric disorder typically accompanied by an extreme sense of fear at the time of trauma occurrence, with characteristic re-experiencing, avoidance, and hyperarousal symptoms in the months and years following the trauma. PTSD has a prevalence of ~6% but can occur in 25–35% of individuals who have experienced severe psychological trauma, such as combat veterans, refugees, and assault victims [1–3]. The differential risk determining those who do versus those who do not develop PTSD is multifactorial [4–7]. It is in part genetic, with at least 30–40% risk heritability for PTSD following trauma [8–10], and in part depends on past personal history, including adult and childhood trauma and psychological factors which may differentially mediate fear and emotion regulation. Additionally, considerable evidence now supports a model in which PTSD can be viewed, in part, as a disorder of fear dysregulation. This is advantageous because the neural circuitry underlying threat and fear-related behaviors in mammals, including the amygdala–hippocampus–medial prefrontal circuit, is among the most well-understood behavioral circuits in neuroscience [11–14]. Further, the study of threat behavior and its underlying circuitry has led to some of the most rapid progress in understanding learning and memory processes.

Although the amygdala and other subcortical regions are perhaps best understood with relationship to threat processing across species, burgeoning evidence has provided substantial support for the role of different regions of the prefrontal cortex (PFC) in particular in regulating the encoding of threat-related behaviors across species and the emotion of fear in humans. Furthermore, the PFC has a critical role in threat inhibition and extinction, as well as in processes such as emotion regulation and avoidance.

In contrast to the promise of current scientific approaches, in the clinic PTSD remains very difficult to treat [15, 16]. The best current treatments are in the form of exposure-based cognitive-behavioral therapies, which are thought to act on the neurocircuitry of threat extinction, in particular through the PFC. The medication treatments for PTSD are primarily limited to traditional serotonin and norepinephrine reuptake inhibitors, which are used for a broad range of depression and anxiety disorders. Advances in understanding the neural circuit of regulation of threat, fear, and PTSD symptoms may lead to novel and more robust treatment approaches.

This review aims to synthesize our current understanding of the role of the PFC in threat behaviors and threat-related emotional processing, and the role of multiple PFC subregions in PTSD. As acknowledged, this line of research is relevant to the treatment of disorders characterized by fear, such as PTSD. However, in line with the two-system view of fear and anxiety [17] and in order to not make assumptions about emotional states, the term “threat” will be used when referring to the behavioral, psychophysiological, or neural outcomes of conditioning research. The term “fear” will be reserved for describing studies in which the subjective emotion of fear was assessed or discussing the emotion more generally.

## Nonhuman animal research on threat processing

The medial prefrontal cortex (mPFC) of the rodent regulates a balance between goal-oriented and habitual behaviors [18, 19]. The mPFC receives massive inputs from subcortical structures, including the amygdala, hippocampus, ventral striatum, hypothalamus, periaqueductal gray, and cerebellum, among others, that allow it to integrate the behavioral state of the animal and adjust behavioral decisions on a moment-to-moment basis. One of the most important mPFC functions is to integrate information about potential threats in the environment with other organismal drives to determine behavioral outputs [20].

Decades of basic research on the mPFC in rodents indicate that it plays a key role in the expression and storage of the Pavlovian threat response and the establishment of threat-related extinction memories [21]. Technological advances have evolved from lesion and pharmacologic studies to experiments utilizing circuit-perturbing and single-cell approaches, which are beginning to provide data at the cell-type resolution for the role of this critical structure in the threat response. Below, we will briefly review the anatomy of the rodent mPFC, the data implicating mPFC circuitry in the threat response and in threat extinction, molecular changes in mPFC cell types with threat acquisition and extinction, and future steps in these lines of research.

### Anatomy of rodent mPFC

**Fig. 1:**
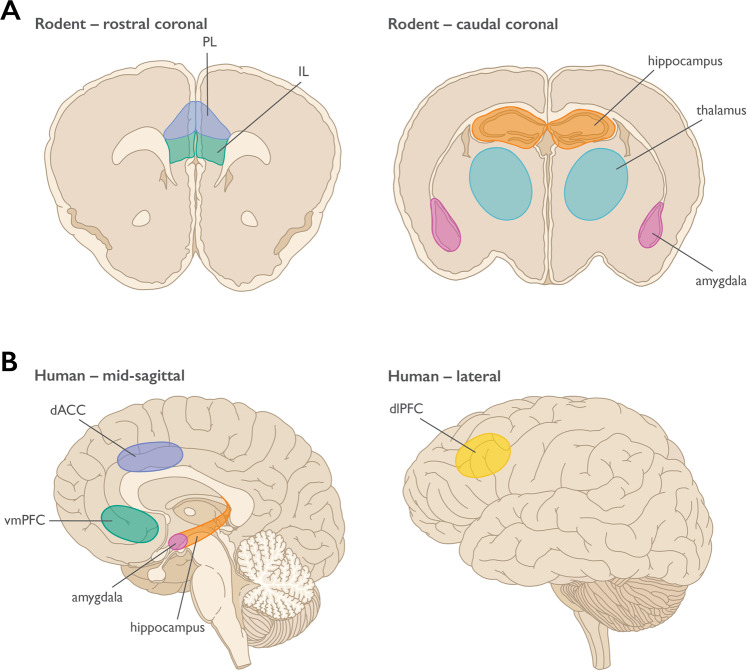
Threat regulatory neurocircuitry across species. **a** Rodent anatomy highlighting regions involved in threat learning, extinction, avoidance, and the contextual modulation of threat expression; **b** Human anatomy highlighting regions involved in threat learning, extinction, avoidance, cognitive regulation, and the contextual modulation of threat expression. PL = prelimbic cortex, IL = infralimbic cortex; dACC = dorsal anterior cingulate cortex, vmPFC = ventromedial prefrontal cortex, dlPFC = dorsolateral prefrontal cortex.

Like most cortical regions, the mPFC is a multi-layered structure of heterogeneous cell types, composed of excitatory pyramidal neurons, inhibitory interneurons, and support cells. Beginning with Brodmann, there have been debates about the existence and location of the mPFC in rodents due to the lack of a prominent granular layer [22; see Preuss and Wise, this issue]. Cross-species comparisons can be more easily made with respect to connectivity patterns [23]. The rodent mPFC is generally considered to consist of the medial precentral area (Fr2), the dorsal anterior cingulate cortex (dACC), prelimbic cortex (PL), and infralimbic cortex (IL) [24] (see Fig. 1a).

For the purposes of this review, we will focus on rodent PL and IL, although recent work has implicated dACC in observational threat pathways, which may be relevant to PTSD from witnessed trauma [25]. Histologically in the mouse, PL and IL differ in the thickness of layer II/III and the prominence of layer separations between superficial II/III and layer V; however, this boundary is not easily demarcated [26, 27]. Both PL and IL receive cortical input, as well as unidirectional projections from the hippocampus, mainly CA1 and subiculum [24]. Projections to the amygdala are bidirectional, although there are differences in the projection patterns of PL and IL to the amygdaloid complex, and there is some controversy about whether PL and IL synapse onto functionally different cell types [28, 29]. Although there is some overlap in projection patterns, IL projects most heavily to lateral septum, bed nucleus of the stria terminalis, amygdala, hypothalamus, and brainstem, while PL sends more projections to insular cortex, nucleus accumbens, thalamus, and raphe nuclei [29]. The differences in these projection patterns suggest diverging functional roles for these adjacent structures.

### Evidence for PL/IL distinction

For the past 20 years, there has been an extensive, although debated, literature showing differential roles for PL and IL in threat conditioning and threat extinction [21]. The first study to demonstrate a role for the rodent ventromedial prefrontal cortex (vmPFC) came from Morgan et al., who lesioned the mPFC [30]. A follow-up study demonstrated that more dorsal areas of the mPFC affected threat learning, while more ventral mPFC was required for threat extinction [31]. Quirk et al. supported this result when they [32] performed electrolytic lesions of the rat vmPFC and assessed threat extinction memory. They found that lesions that included caudal IL ablated threat extinction memories, while those that excluded the area had no effect. Pharmacological inactivation of PL and IL with agents such as the GABA agonist muscimol further suggested opposing roles for these structures in threat conditioning and threat extinction, respectively [33]. However, these results have not been universally reproduced [34, 35]. More recent studies from the Quirk laboratory have used optogenetics to drive or inhibit activity in excitatory IL neurons during threat extinction. These data suggest that IL neurons are necessary for encoding threat extinction memories but may not be necessary for threat extinction memory storage or retention [36]. These findings also suggest that the threat extinction memory trace may be represented by different cell populations over time. Indeed, it has been known that the threat memory is likely constituted by a distributed network of cells across a range of brain regions. Inputs to the mPFC likely help to drive evolution of the memory trace over time.

### Modulation of mPFC by subcortical structures

Because the mPFC must guide behavior on a moment-to-moment basis, it needs to receive a constant stream of information from subcortical structures and send out a coordinated response. The mPFC receives dense innervation from many subcortical structures, but we will focus here upon three crucial inputs: the hippocampus, amygdala, and thalamus. The canonical role of the hippocampus in threat circuitry is to encode context-specific information of a threat trace, as it is crucial for an organism to be able to distinguish threats as belonging to a particular context. The hippocampus itself appears to have a dorsal-ventral functional gradient, with the dorsal hippocampus encoding context more specifically, while the ventral hippocampus (vHPC) includes affective information as well [37].

The vHPC sends dense direct projections to the mPFC from CA1, but also bidirectional disynaptic indirect connections to the mPFC through the reuniens nucleus of the thalamus and the perirhinal cortex [38]. Lesion studies of the hippocampus suggest a critical role in context processing [39]. Reversible inactivation of the dorsal hippocampus, through either pharmacologic or chemogenetic means, interferes with context-specific information of a threat memory [40, 41]. Inhibition of double-projecting vHPC neurons to the mPFC and basolateral amygdala (BLA) interferes with contextual threat recall [42] and disconnection of the vHPC from the mPFC interferes with renewal of threat memories, a context-dependent process [43]. Activity-tagging coupled with optogenetic inhibition suggests that threat conditioning and extinction memories exist in separate populations of neurons within the hippocampus [44], and the hippocampus may influence mPFC activity through feed-forward inhibition mechanisms through parvalbumin interneurons [45]. In return, the mPFC appears to suppress expression of erroneous contexts in a “top-down” manner through a disynaptic pathway through the reuniens nucleus of the thalamus [46]. In addition, there may be more routes of information flow from the PFC to the hippocampus, including direct routes from the nearby anterior cingulate [47].

The amygdala communicates the salience of the threat cue to the mPFC (see Murray and Fellows, this issue, for further discussion of amygdala-PFC interactions). For thirty years, the amygdala has been implicated in both threat learning [48] and threat extinction [49] processes. The BLA sends bidirectional projections to the mPFC [50]. There is evidence to suggest that there is a dorso-ventral topographic segregation of BLA input to the mPFC; more dorsal projections (to PL) encode threat-stimulating information while more ventral projections (to IL) encode threat extinction-related information [51]. Synaptic connections between PL neurons and BLA inputs also strengthen in response to stress, in part through endocannabinoid-mediated mechanisms [52]. Projection neurons within the BLA exhibit plasticity when conditioned stimulus-unconditioned stimulus pairings occur and convey this information to the mPFC.

Finally, nuclei within the thalamus help bind threat memories to context and facilitate shifts in the mPFC threat memory trace over time. The reuniens nucleus of the thalamus coordinates oscillatory synchrony between the mPFC and the vHPC, which is necessary for proper contextual representation of threat memories [46, 53]. The paraventricular nucleus of the thalamus plays a crucial role in the encoding of threat memories over time [54] and appears to be necessary in shifting the temporal nature of how the mPFC encodes threat memories [55].

### Molecular pathways in rodent mPFC

At the molecular level, threat conditioning and extinction are associated with epigenetic, transcriptional, and translational changes that likely modify synaptic weights and cell firing properties that persistently alter circuit function. Introduction of the translational inhibitor anisomycin, either intraventricularly or into the mPFC, causes a failure to retain threat extinction memories. This suggests that translation of new protein is necessary for the formation of a novel threat extinction memory [56]. Threat conditioning and threat recall are associated with unique, cell-type-specific transcriptional changes that persist for weeks after initial training [57]. Threat extinction also requires transcriptional processes within IL: injection of an inhibitor of PARP-1, a gene involved in ADP-ribosylaton that is necessary for transcription, into mPFC impairs contextual threat extinction [58].

The BDNF-TrkB neurotrophic factor pathway has also been extensively studied with regards to mPFC and memory formation in mPFC. Expression of *Bdnf* in PL is necessary for consolidation of cued threat conditioning [59], while infusion of *Bdnf* into IL after threat acquisition is sufficient to diminish threat responses in the absence of extinction training [60]. Threat extinction is also associated with epigenetic modification. In the IL, threat extinction is associated with acetylation of histones near the *Bdnf* locus [61], changes to the p300/CBP complex (PCAF) [62], as well as deposition of DNA-modification marks such as 5-hydroxymethylcytosine and N6-methyl-2’deoxyadenosine (m6dA) near loci of activity-dependent genes such as *Bdnf*.

Additionally, inhibition of PCAF in IL was shown to interfere with threat extinction [62]. Recently, Li et al. [63], have shown that knockdown of *N6amt1*, the gene responsible for m6dA deposition, within IL, blocks changes to the m6dA mark at the *Bdnf* promoter in vivo and impairs threat extinction retention. These findings suggest that alterations in m6dA deposition are necessary for the formation of threat extinction memories within IL [63]. These findings also strongly support the hypothesis that threat extinction memory requires epigenetic changes within IL. Our understanding of the molecular changes that occur within the mPFC during threat-related processes are still in their infancy. Gene expression changes are unique to cell type, and cell-type-specific investigations of mPFC in threat conditioning and extinction are just beginning.

### Stress and threat reactions

One potential factor that alters the ability to control emotional responses via altering PFC function is stress (for review, see 64,65, Kalin and Barbas, this issue). Studies in animal models have shown that acute stress leads to changes in neuronal signaling that impair function in the dlPFC [64] and IL cortex [66]. These changes are proposed to be due to the impact of increased catecholamines, in particular noradrenergic and dopaminergic signaling, on PFC neuronal activity with even relatively mild acute stress exposure [64, 65, 67]. Stress also impacts signaling within the amygdala. Noradrenergic signaling from the locus coeruleus to the amygdala was recently shown to be necessary to produce the immediate extinction deficit, an impairment in extinction learning that occurs soon after fear learning and is thought to be related to the stress of the fear learning process [68]. Activity of CRF-expressing neurons within the CeA was also recently shown to contribute to this phenomenon [69]. In rodents, chronic stress also impacts neural activity in both PL and IL cortex [70] and leads to structural changes in IL cortex [71]. One consequence of stress-related PFC impairment is enhanced threat learning and impaired extinction retention in rodent models [66, 70].

In the next section we will explore the role of PFC in human threat processing research, from acquisition and encoding of threat, to its extinction and extinction recall. We will also further integrate additional findings with regards to other threat and avoidance behaviors in response to threat stimuli and the impact of stress on the PFC. Finally, we will examine how these different brain regions and behaviors are dysregulated in threat-related disorders such as PTSD.

## Preclinical human threat processing research

### Threat learning

**Fig. 2:**
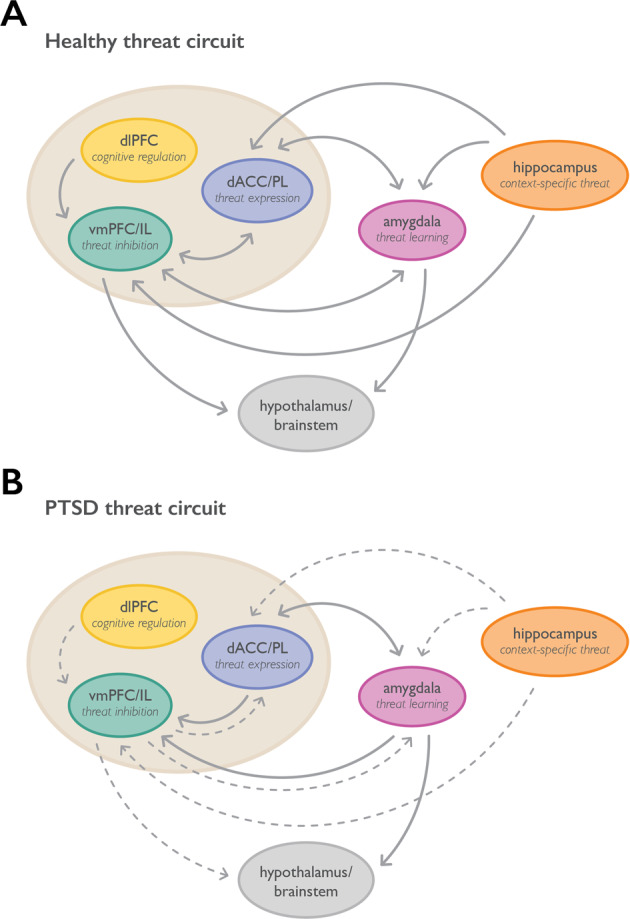
Functional connections in threat circuitry in health and PTSD. **a** Healthy Threat Circuit. Regions involved in threat learning and the control of threat reactions via extinction, context, avoidance, or cognitive regulation. In healthy individuals the coordination of this circuit enables adaptive threat expression. **b** PTSD Threat Circuit. The dlPFC, vmPFC/IL, and hippocampus show impaired functioning with PTSD, whereas the amygdala and dACC/PL are enhanced. Disrupted connections between regions are indicated by dashed lines. The disrupted threat circuit with PTSD results in maladaptive threat expression. Prefrontal cortex regions are highlighted within the beige circle. Terms for animal/human homologous regions are in the same circles. PL = prelimbic cortex, IL = infralimbic cortex, dACC = dorsal anterior cingulate cortex, vmPFC = ventromedial prefrontal cortex, dlPFC = dorsolateral prefrontal cortex.

Perhaps it is not surprising, given the extensive research with nonhuman animals, that research in humans confirms a role for the amygdala and PFC in threat learning (see Fig. 1b and Fig. 2a). The role of the amygdala was first demonstrated in patients with amygdala damage. Relative to healthy controls, both bilateral [72] and unilateral [73] amygdala damage resulted in impaired conditioned responses, as measured by the skin conductance response (SCR). However, these patients were able to verbally report the contingency between the conditioned stimulus and shock after the procedure, which was impaired in patients with hippocampal damage whose amygdala was intact [72, 74]. These findings suggest that the amygdala is only critical for the implicit, physiological expression of threat learning in humans, with conscious knowledge about the threatening nature of stimuli in the environment remaining intact, despite amygdala lesions. Furthermore, these findings demonstrate that there are additional brain regions that are critical for the expression of the subjective fear and threat responses, including PFC areas that are discussed in more detail below.

Consistent with these early patient studies, functional magnetic resonance imaging (fMRI) studies soon followed that showed increased blood oxygenation level dependent (BOLD) signal in the amygdala to a conditioned stimulus (relative to stimulus never paired with shock) [75, 76], and the magnitude of this BOLD response was correlated with the strength of the conditioned response [76]. Interestingly, this differential amygdala BOLD response was only apparent in the early stages of threat conditioning. This finding is somewhat surprising given rodent research showing long-lasting changes in the amygdala lateral nucleus with threat learning. However, there is electrophysiological evidence in rodents showing that a subset of lateral nucleus amygdala neurons respond preferentially during initial learning [77], and there is greater responding overall at this time. It may be the case that BOLD changes in the amygdala can only be observed at time windows when there are larger populations of neurons responding, such as initial learning. One major limitation of fMRI for investigations of amygdala function in humans is that it is a relatively coarse measurement. Although the spatial resolution of standard BOLD imaging is generally 3 mm, in practice, with spatial smoothing and group averaging, the actual resolution is greater than 10 mm, which covers a substantial portion of the amygdala (which is slightly more than 1000 mm^3^ in humans) and makes it very difficult to detect discrete responses in amygdala subnuclei. The challenges of using BOLD imaging to study the human amygdala is reflected in recent meta-analyses of fMRI threat learning studies, which fail to find BOLD changes in the amygdala [78], in spite of its critical role in threat learning in rodent models and patient studies.

In contrast to difficulties in detecting BOLD changes in the amygdala during threat learning, meta-analyses and individual studies reliably show activation in a number of other brain regions, including the insula cortex, which is linked to physiological arousal responses [79], the striatum, and the dACC (e.g., [76, 78, 80]). The dACC is a prefrontal region that is proposed to be the human homolog of the PL cortex in rodents [80]. As discussed earlier, the PL in rodents has been suggested to play a role in the expression of threat learning via projections to the basolateral amygdala, with stimulation of this region increasing conditioned freezing and inactivation reducing it.

In rodents, the PL and IL cortex are located adjacent to one another in the mPFC. In primate models, however, the PL and IL are farther apart. The primate PL cortex is thought to be divided into rostral and caudal regions with different connectivity patterns. The rostral region is thought to be more similar to the PL in rodents, with the dACC being the human homologue for that region [23, 81]. Consistent with this suggestion, Milad et al. [80] found that both cortical thickness, and BOLD response magnitude to a conditioned stimulus in this region, were correlated with the strength of the conditioned response as measured with SCR in humans.

Although the basic circuitry of threat learning seems to be preserved across species, a primary difference between humans and other animals is that humans, more often than not, learn about threats in the environment via social interactions. For example, children learn to fear germs by being told about their existence and observing others engaging in actions attempting to avoid them. This ability is adaptive in that humans do not need to be physically harmed to learn about threats in the environment. It can also be maladaptive in that we can develop robust fears for events that are imagined and anticipated but never actually experienced, contributing greatly to human anxiety and fear-related disorders. To what extent do the brain systems involved in threat learning from direct experience, that have been investigated in rodent models, map onto socially acquired, imagined threats in humans?

To address this question, brain imaging and patient studies have examined threat learning through verbal instruction (e.g., being told a blue square predicts a shock, and then being shown a blue square) or observation (e.g., watching someone else receive a shock paired with a blue square, and then being shown a blue square). Consistent with Pavlovian threat conditioning, fMRI studies of both instructed and observational threat learning show activation in the amygdala, dACC, and insula [82]. For instructed learning, the amygdala BOLD response is left-lateralized [83], and only patients with left, but not right, amygdala damage show impaired physiological evidence of threat learning, perhaps because of the verbally mediated nature of this learning [84]. In contrast, observational learning results in increased bilateral BOLD signal in the amygdala, both when observing someone else receiving a shock paired with a conditioned stimulus (learning), and when viewing the conditioned stimulus afterwards (test). In addition, during observational learning, activation in a rostral mPFC region that has been implicated in mentalizing about others is correlated with the strength of the learned threat response as measured by SCR [85], and learning is stronger with greater empathy for the person being observed [86]. These results suggest that while the social learning of threat may engage unique neural circuitry due to the nature of the learning, it also takes advantage of the phylogenetically older mechanisms of Pavlovian threat conditioning for threat expression.

### Threat extinction

Much like threat learning, neuroimaging studies of threat extinction in humans have identified brain regions that parallel those involved in extinction in rodents (see Figs. 1b and 2a). The vmPFC is proposed to be the homologue for the IL in rodents [87] and serves to inhibit threat responses produced by the amygdala. There is consistent evidence of increases in BOLD signal in the vmPFC during extinction learning [88–90] and recall [89–92] (for review, see [93–95]). Further, the degree of activation of the vmPFC has been shown to be positively correlated with the degree of extinction, or extinction retention, as measured by SCR [89, 90], consistent with the suggested role of the IL in extinction in rodent models.

Brain morphology studies also point to the human vmPFC being involved in extinction. Milad et al. [96] found vmPFC thickness to be positively correlated with extinction recall. Specifically, greater thickness was associated with smaller SCR to the conditioned stimulus during extinction recall, suggestive of better extinction recall (see also [97]). Subsequently, Winkelman et al. [98] examined the relationship between vmPFC thickness and extinction learning, rather than recall, and found similar results. Greater vmPFC thickness was associated with smaller differential SCR during early extinction learning, suggestive of better extinction learning.

### Targeting extinction with neuromodulation, neuroplasticity, and context modulation

One drawback of these MRI studies, however, is that they are correlational in nature. Unlike research conducted in rodents, specific brain regions in humans cannot be lesioned or tagged, nor can regions that are not on the surface of the brain be disrupted. Researchers are able, however, to stimulate or disrupt surface frontal regions of the brain in humans using non-invasive devices. For example, transcranial direct current stimulation (tDCS) applies a low-intensity current through two electrodes attached to the scalp, and transcranial magnetic stimulation (TMS) delivers an electric current through a coiled wire placed on the scalp, creating a magnetic field across the skull. Both of these strategies are thought to modulate neuronal activity in the human brain.

Using these techniques, a few recent brain stimulation studies [99–103] have been conducted in humans to probe the role of the vmPFC in extinction. For example, Dittert et al. [100] administered tDCS via bitemporal electrodes aimed at the vmPFC prior to and during extinction and found that tDCS, relative to sham, stimulation resulted in faster early extinction learning. Similarly, Raij et al. [102] found that TMS, during extinction learning, to an area of the frontal cortex functionally connected to the vmPFC (i.e., the left lateral PFC), but not to an area of the frontal cortex thought to be unconnected to the vmPFC, led to enhanced extinction recall. Although these studies provide some insight into the role of the vmPFC in extinction, given the location of the vmPFC and the fact that tDCS and TMS are applied externally, it is difficult to be certain that the vmPFC in particular was stimulated in these studies.

Consistent with animal models of extinction circuitry, the vmPFC interacts with other regions such as the amygdala and hippocampus to modulate threat responses during extinction. From rodent research showing that intra-amygdala infusion of the NMDA receptor agonist d-cycloserine, which enhances NMDA-dependent plasticity, facilitates extinction learning, and successful translation of this work to humans (see [104] for review), we know that the human amygdala plays a role in extinction learning. Human imaging studies, however, have been less consistent with finding changes in BOLD signal in the amygdala during extinction [76, 88, 90, 95, 105]. Much like with threat acquisition, it may be that the involvement of the amygdala in extinction is more subtle and difficult to detect using standard fMRI techniques [106]. Nonetheless, imaging research does point to changes in the relationship between the PFC and amygdala during extinction. Connectivity analyses have demonstrated functional coupling between the mPFC and amygdala during extinction learning [107], and vmPFC and amygdala during extinction recall [89, 108].

Also consistent with animal models (e.g., [40]), research suggests that the hippocampus is involved in contextual modulation of extinction and works in concert with the PFC during contextual extinction learning. One of the first studies demonstrating hippocampal involvement in extinction showed that patients with damage to the hippocampus failed to show contextually modulated reinstatement of conditioned responses following extinction [74]. Brain imaging studies of the contextual modulation of extinction typically manipulate the visual background during extinction and report hippocampal activation during extinction recall [89, 91, 92]. Importantly, functional connectivity analyses also suggest coupling of the PFC and hippocampus during contextual extinction learning [107] and recall [89].

Sleep is another factor that has been shown to modulate threat control in humans. Sleep has been shown to enhance both threat learning, and the generalization of extinction learning in humans and other animals. The documented role for sleep in memory consolidation is proposed to extend to both threat memories and extinction memories. Which of these competing memory representations is selectively strengthened depends on contextual factors such a recency of learning and replay [109]. Because of evidence for sleep’s modulation of extinction learning across species, it has been suggested that disruptions of sleep following acute trauma, or predating the traumatic experience, may contribute to the etiology or perpetuation of PTSD [110].

### Avoidance/active coping

Another method of reducing conditioned threat reactions is through active avoidance or coping. Initial rodent research on the neural circuitry of active avoidance found that while the passive expression of conditioned threat responses engages a pathway from the lateral nucleus to the central nucleus, when the animal engages in an action to avoid the unconditioned stimulus, projections from the lateral nucleus to the basal nucleus to the nucleus accumbens are involved. However, in order for the animal to produce an avoidance action, conditioned freezing must be inhibited which requires the IL cortex, much like in the expression of extinction (see [111] for a review).

One benefit of avoidance learning over extinction for controlling threat reactions is that avoidance learning results in a persistent reduction in the passive conditioned response, even when the avoidance action is no longer available [112]. This is in contrast to extinction in which the conditioned response often returns through spontaneous recovery, renewal, or reinstatement. Both the acquisition of avoidance, and the reduction of the persistent conditioned threat reaction following avoidance learning, are blocked by the injection of protein synthesis inhibitors into the IL. This indicates that plasticity in the IL is critical for the persistent reduction of conditioned responses with avoidance [112]. Mirroring these findings, studies have shown that previous history with escapable shock results in a lasting reduction of the conditioned response, and this effect is eliminated with IL inactivation [113].

In humans, there is evidence that both avoidance learning and history with escapable shock can persistently reduce conditioned threat actions as measured with SCR, even when no avoidance action is available [114, 115]. However, in order to persistently diminish threat conditioned responses in humans, avoidance actions need to be learned through trial and error and there needs to be a subjective sense of control over the unconditioned stimulus during learning [115]. Simply providing the option of an action to avoid the unconditioned stimulus yields no lasting reduction of conditioned responses when the avoidance action is no longer available, and in fact can increase them by preventing extinction learning (called “protection from extinction”, [116]). Consistent with the circuity of avoidance learning detailed in rodent models, trial-by-trial avoidance learning yields increased BOLD activation in the vmPFC and ventral striatum, relative to standard extinction [114], suggesting the brain mechanisms of active coping are preserved across species.

### Emotion regulation

Although extinction and active coping can be investigated across species, humans have the unique ability to use cognitive strategies to alter emotional responses, such as responses to fear provoking stimuli (for review, [117]). One common emotion regulation strategy is cognitive reappraisal. This strategy involves reframing thoughts (also called “appraisals”) about a stimulus in order to change the emotional response that that stimulus evokes. Emotion regulation strategies can be employed with the goal of either upregulating (i.e., increasing) or downregulating (i.e., decreasing) emotions. Here, the primary focus is on data related to downregulating negative emotions, fear in particular, as these data are most relevant to PTSD and its treatment. We highlight reappraisal, which is proposed to be similar to cognitive restructuring in clinic. Neuroimaging research has provided insight into the brain regions involved in emotion regulation in humans. Studies to date suggest that emotion regulation strategies aimed at downregulating negative emotions engage cognitive control regions of the PFC, which then modulate the amygdala via various potential pathways to influence negative emotional responses.

The most recent meta-analysis of fMRI studies of emotion regulation [118] found that all strategies aimed at downregulating negative emotions were collectively associated with increased BOLD signal in the following areas: ventrolateral prefrontal cortex (vlPFC), dorsolateral prefrontal cortex (dlPFC), and dorsomedial prefrontal cortex (dmPFC). While these were the largest areas of convergence, activation was also found in other areas (i.e., the bilateral inferior parietal lobule, supplementary motor area, pre-supplementary motor area, left middle temporal gyrus, and posterior cingulate gyrus). These findings were relatively consistent with a prior meta-analysis [119], with the exception that the prior meta-analysis also found decreased BOLD signal in the amygdala and parahippocampal gyrus, consistent with the notion that cognitive control regions of the PFC modulate amygdala activity during emotion regulation. One potential reason for differing results across these two meta-analyses may be differences in the studies examined and proportions of various emotion regulation strategies included. There is some evidence that different emotion regulation strategies may recruit distinct brain regions. For example, Dörfel et al. [120] found that some emotion regulation strategies are associated with reduced activity in the amygdala, whereas others are not.

Nonetheless, the majority of imaging research to date on emotion regulation focuses on the strategy of cognitive reappraisal. Meta-analyses of cognitive reappraisal alone have consistently found increased BOLD signal in the dlPFC, vlPFC, and dmPFC [93, 121, 122] and decreased BOLD signal in the amygdala [93, 121]. The dlPFC is thought to be an important driver of emotion regulation and hypothesized to be involved in the manipulation of appraisals of stimuli in working memory [121–123]. The vlPFC is hypothesized to support choosing and inhibiting appraisals of stimuli [121, 124, 125] or potentially may signal salience and the need to reappraise [122]. Finally, the dmPFC is hypothesized to support abstracting affective meaning of stimuli or the processes of self-reflecting and identifying one’s own affective reactions to stimuli [121, 126–130].

Two hypotheses have been proposed for how these cognitive control regions of the PFC (i.e., the dlPFC, vlPFC, and dmPFC) influence the amygdala: (1) that they engage the vmPFC which then modulates the amygdala, similar to the neurocircuitry of extinction [93, 131, 132], and (2) that they modulate lateral temporal areas associated with semantic and perceptual representations, which then indirectly influence the amygdala [121].

In support of the first hypothesis, anatomical research in nonhuman primates shows that connections between the lateral PFC and amygdala are sparse relative to the vmPFC and amygdala (e.g., [133, 134]). Additionally, a study of cognitive reappraisal of threat conditioned stimuli by Delgado et al. [131] demonstrated increased BOLD signal in the dlPFC and decreased BOLD signal in the amygdala, but also changes in vmPFC activity that mirror those that occur during extinction. Specifically, increased BOLD signal in the vmPFC after successful cognitive reappraisal was observed. Further, connectivity analyses [131, 132], a meta-analysis conducted by Diekhof et al. in 2011, and more recent dynamic causal modeling of cognitive reappraisal data [135], also support this hypothesis.

In contrast, two more recent meta-analyses favor the second hypothesis [121, 122]. Specifically, in addition to increased activation of the cognitive control regions of the PFC mentioned above and modulation of the amygdala, Buhle et al. [121] report increased activation of the lateral temporal cortex during cognitive reappraisal. Notably, they did not observe increased activation of the vmPFC. There are many possible reasons for these differences in findings (see [121, 136, 137] for discussion), however, one possibility is that it has to do with the type of cognitive reappraisal procedure used. The study by Delgado etv al. [131] involved cognitive reappraisal of threat conditioned stimuli, whereas most of the studies included in the Buhle et al. [121] meta-analysis involved cognitive reappraisal of negatively-valenced photos. It could also be that the vmPFC is involved in cognitive reappraisal for some individuals more so than others. For example, the nature of vmPFC involvement during cognitive reappraisal has been observed to vary as a function of psychiatric symptoms [138, 139].

To date, the majority of studies on brain regions involved in emotion regulation have examined fMRI data. To our knowledge, only one study has examined brain morphometry in relation to cognitive reappraisal task performance [97] and this study did not find a relationship between success of reappraisal and dlPFC, vlPFC, or vmPFC cortical thickness. Although there do not seem to be structural changes related to the successful cognitive reappraisal, there is one lesion study [140] that supports a role of the dlPFC in cognitive reappraisal. This study examined individuals with dlPFC lesions and found that they showed impaired ability to reappraise threat conditioned stimuli as indexed by poorer subjective fear outcomes, compared to matched controls. Additionally, a few brain stimulation studies have been conducted to probe the role of the PFC in emotion regulation. In this case, given the lateral location of the cognitive control regions of the PFC implicated in emotion regulation, tDCS or TMS can be used to target these regions. However, results of these studies have been inconsistent. While some studies have found that tDCS over the dlPFC or vlPFC enhances cognitive reappraisal [141–143], others have not [143, 144].

### Stress and threat control

As the discussion above indicates, there are several techniques that can be used to control learned threat reactions in humans when they are no longer adaptive. However, as outlined earlier, stress, both chronic and acute, can impact the function of PFC and subcortical regions implicated in threat control. In humans, experimental studies of chronic stress are not possible due to ethical concerns; however, there is evidence that a history of childhood abuse is correlated with reduced gray matter volume in PFC regions, including the vmPFC and orbital frontal cortex [145]. In addition, mild acute stress in humans impairs the efficacy of previously acquired cognitive reappraisal strategies in reducing conditioned threat and subjective fear [146] and results in enhanced spontaneous recovery following extinction training [147]. These latter findings suggest that even when threat control techniques are successfully learned, relatively mild acute stress may impair the ability to express this learning by impacting the function of PFC inhibitory circuits.

## Role of PFC in PTSD

The above evidence suggests that stress, in particular, is associated with altered PFC function and its role in regulating subcortical emotional responses. PTSD is among the most well-understood, prevalent, and medically significant stress-related disorders. A fairly large set of studies now supports a clear role for altered PFC structure and function in PTSD and related disorders (see Fig. 2b).

### Structural imaging in PFC and PTSD

Related to stress exposure, independent of PTSD, a number of studies have identified smaller volumes in PFC and decreased structural connectivity between PFC and subcortical areas as a function of violence and trauma exposure. In a prospective study of Israeli soldiers, Admon et al. [148] used diffusion tensor imaging (DTI) pre- and post-military service, reporting that soldiers with decreased hippocampal structural connectivity with the vmPFC had a more maladaptive response to stressful military service. In a small study of victims of urban violence, Rocha-Rego et al. [149] found significant reductions in gray matter volume in the ventral premotor cortex and in the pregenual ACC as a function of civilian violence. Furthermore, in a moderately large sample of post-9/11 veterans, Clausen et al. [150] found that higher combat exposure uniquely related to lower cortical thickness in the left prefrontal lobe; and that, overall, combat exposure, PTSD, and head injuries differentially relate to alterations in cortical thickness.

A number of studies have also found decreased gray matter volumes related to traumatic stress symptoms. In development, with a study of ~50 youth, Keding and Herringa [151] found that those with PTSD had reduced gray matter volume in anterior vmPFC, which inversely correlated with PTSD duration. They suggest that pediatric PTSD is associated with abnormal structure of the vmPFC, possibly related to disrupted extinction and contextual gating of fear. Similarly, in a study of over 100 participants, it was found that maltreated youth with PTSD demonstrated decreased right vmPFC volumes compared to both maltreated youth without PTSD and nonmaltreated healthy controls [152]. Similar findings have been found in adult samples. A study of 85 veterans suggested decreased structural volumes of vmPFC and ACC in those with PTSD compared to controls [153] and a smaller study of 28 veterans found smaller subgenual cingulate volumes compared to controls, in addition to a number of other limbic region structural abnormalities [154].

A number of studies have also examined structural integrity of white matter tracts, including connections to and from the PFC. Koch et al. [155] used DTI to show decreased integrity of the uncinate fasciculus tract, connecting the vmPFC to multiple subcortical, limbic regions including the amygdala, in patients with PTSD. In addition, using DTI, Fani et al. [156] found that civilians with PTSD had decreased structural connectivity via the cingulum bundle, which supports the hippocampus-dACC pathway. They suggest that altered hippocampus-ACC connectivity may represent a highly salient intermediate neural phenotype for PTSD. Further analyses of this cohort found that individuals with the “risk” allele of the *FKBP5* genetic biomarker, associated with childhood maltreatment and PTSD risk, also had decreased cingulum structural integrity.

Some of these structural changes may occur quite rapidly in the aftermath of trauma—or may be preexisting and predispose some individuals to a greater risk of PTSD following trauma. Using DTI and structural imaging in the weeks following trauma exposure, research has shown that reduced fractional anisotropy of the uncinate fasciculus at around the time of trauma predicted greater PTSD symptoms (in particular posttraumatic anhedonia) at 12 months post-trauma. Furthermore, as the traumatized participants were followed over time, increased gray matter volume of the vmPFC was also associated with reduced trauma-related symptoms over the 12 months following trauma [157]. In another study recruiting patients after trauma exposure with mild traumatic brain injury, smaller cortical volumes of superior frontal cortex and rostral and caudal cingulate at 2 weeks after trauma exposure contributed to the prediction of increased likelihood of 3-month PTSD diagnosis in multivariable models incorporating other established risk factors [158].

As with many other types of human data, sample size often limits interpretation, as small sample sizes are subject to both false positive and false negative biases. Therefore, as more studies have been performed of PTSD and structural imaging, much larger analyses can be performed via meta-analyses of multiple datasets. A recent meta-analysis confirmed, using voxel-based morphometry, that there were prominent volumetric reductions in the mPFC, including the ACC, when examining over 80 different MRI studies in PTSD compared to depression [159].

In addition, one of the largest meta-analyses to date of structural cortical volumes from the ENIGMA-PGC-PTSD workgroup, compared 1379 PTSD patients to 2192 controls without PTSD. A primary finding was that volumes of left and right lateral orbitofrontal gyri were significantly smaller in PTSD patients than controls and were negatively correlated with symptom severity. Together, these findings indicate that cortical volumes in PTSD patients are smaller in prefrontal regulatory regions, consistent with preclinical work suggesting a critical role for orbital frontal PFC regions in recovery and extinction of threat behaviors [160].

### Functional imaging in PFC and PTSD

It is thought that fMRI, including both emotional and cognitive task-based MRI, as well as resting state MRI may be more sensitive to pathology-related functional activity. However, it can also be criticized as there are many processing steps and the data are essentially subtractions and comparisons between different tasks, timepoints, and individuals, making interpretation often more complex than structural MRI. Nonetheless, many of the same themes with regards to the role of PFC in PTSD are found with fMRI, complementing the above structural MRI findings as well as the healthy human and preclinical data.

While many fMRI studies of PTSD have had quite limited sample sizes, some are particularly informative. Script-driven imagery with participants reading or listening to scripts of their prior trauma experiences or other stressful vignettes has been a powerful probe for determining differential brain activity in PTSD. For example, in a case-control twin study with 26 male identical twin pairs (12 PTSD; 14 non-PTSD) discordant for PTSD and combat, script-driven imagery fMRI revealed diminished activation in the mPFC during stressful versus neutral imagery in PTSD patients relative to others [161].

Another powerful method for observing brain activity is using a masked emotional probe. In this design, participants are presented with an emotional stimulus for a very brief period of time (i.e., milliseconds) and then it is replaced by a non-emotional stimulus. This minimizes explicit awareness of the emotional stimulus, while still engaging emotional perception and the associated neutral structures. For example, Killgore et al. [162] used a masked emotional probe task and compared adults with anxiety or PTSD to healthy controls. Patients (all groups combined) showed greater amygdala and reduced vmPFC activation compared to controls during the masked fearful faces. Additionally, in a prospective emergency department study following patients longitudinally, Stevens et al. [163] used an unmasked fearful faces design to demonstrate that dorsal ACC activity during presentation of fearful faces predicts ongoing symptom maintenance in the aftermath of acute trauma in civilians. These findings are consistent with some of the findings of Milad et al. [164–166] related to impaired extinction recall, discussed in the next section.

A number of studies have also examined the role of activity within the PFC related to prediction of treatment response. Fonzo et al. [167] reported on fMRI task-based assessment while completing three tasks assessing emotional reactivity and regulation prior to prolonged exposure psychotherapy for PTSD. At baseline, individuals with the greatest symptom improvement with therapy showed, among other findings, greater dorsal PFC and vmPFC activation during emotional conflict regulation. They interpreted these findings to suggest that participants who are most likely to benefit from exposure therapy demonstrate spontaneous activity of PFC when superficially processing threat and adapting to emotional interference. Further analysis of this study suggested that psychotherapy increased lateral frontopolar cortex activity and connectivity with the vmPFC [168]. Additionally, greater increases in frontopolar activation were associated with improvements in hyperarousal symptoms and psychological well-being. Given these findings, the authors argue that frontopolar connectivity with ventromedial regions during emotion regulation is a possible key mechanism of psychotherapeutic improvement in PTSD.

In addition, in a mindfulness-based exposure therapy intervention with a small sample size, King et al. [169] found that posterior cingulate cortex (PCC)-dlPFC functional connectivity was correlated with improvement in PTSD avoidance and hyperarousal symptoms. Overall, they surmised that increased connectivity between PCC-dlPFC brain regions could be related to attentional control and symptom improvement.

It is important to note that the “standard” observation of hyperarousal-related PTSD with increased amygdala activation and decreased mPFC activation during stressful cues seems to be reversed when subjects with PTSD have high levels of dissociative symptoms. Hooper et al. have reported increased mPFC activation in the presence of PTSD with significant dissociative symptoms (e.g., [170]). Dynamic causal modeling has been used to interpret some level of causal and temporal relationship between functionally active brain networks. Nicholson et al. [171] found that PTSD without dissociation was characterized primarily by ‘bottom-up connectivity’ from the amygdala to the vmPFC, whereas PTSD with dissociation had predominant ‘top-down connectivity’ from the vmPFC to the amygdala. The authors suggest that this provides further data supporting a model of enhanced top-down, emotional over-regulation with significant dissociation, in contrast to decreased emotion regulation in the majority of PTSD cases.

Overall, these studies suggest that, in general, decreased vmPFC and dlPFC functional activation to stressful and trauma-related cues, often accompanied by increased dACC activation to such cues, are associated with greater PTSD symptoms and decreased responses to exposure-based therapies. Successful treatment appears to be accompanied by increased activation and functional connectivity of vmPFC/dlPFC regions with other cortical and subcortical areas. Notably, PTSD with significant dissociation seems to have differential connectivity patterns, as recently further demonstrated by Jovanovic et al. [172], and may benefit from distinct approaches to treatment and recovery.

### PFC, threat extinction, behavioral inhibition

Work that has done the most to connect neuroimaging findings in PTSD with both healthy human neuroimaging work and preclinical work on threat acquisition and extinction includes neuroimaging studies specifically focused on extinction of threat cues in PTSD as well as inhibitory learning processes. Milad et al. initially demonstrated some of these effects in PTSD through optimization of a within-scanner threat conditioning and extinction protocol. They found that during extinction recall there was decreased activation of vmPFC and greater activation in dACC in PTSD relative to healthy trauma-exposed participants, which was associated with impaired physiological measures of extinction recall [165]. These data suggest that impairments in PTSD recovery may result, in part, from altered PFC regulation of threat extinction recall. Increased dACC activation during threat expression and extinction and decreased vmPFC activation with extinction recall were replicated in another study by this group examining context modulation in PTSD [166]. The dACC also appears to be associated with more PTSD symptoms at rest. Marin et al. [164] found that dACC resting activity positively correlated with PTSD symptom severity and predicted increased dACC activations during extinction recall.

In addition, Helpman et al. [173] examined threat conditioning and extinction in an fMRI task before and after a course of prolonged exposure treatment for PTSD. They found that PTSD patients had pre- to post-treatment reductions in rostral ACC (rACC) activation during extinction recall, and increases in functional coherence between rACC, vmPFC, and sgACC, suggesting these circuits are modified.

In another approach to this issue, Jovanovic et al. [174] found lower vmPFC activation during a simple stop signal (or “Go/No-go”) task related to behavioral inhibition in civilians with PTSD. Inhibition in this task correlated with a physiological measure of threat extinction in a fear-potentiated startle paradigm outside of the scanner. These data suggested that the same circuits involved in behavioral inhibition appear to be involved in fear inhibition processes during differential threat conditioning and extinction. A follow-up study with a larger sample size suggested that the effects of vmPFC/rACC on behavioral inhibition are moderated by childhood maltreatment effects in participants with PTSD [175].

## Conclusions and future directions

Considerable progress has been made in understanding the role that the PFC plays in rodent threat learning and threat extinction, in healthy human studies of regulation of threat and fear emotion processing, and in the ways these areas may be dysregulated in threat-related disorders such as PTSD. Still, many questions remain. Much data supports a heuristic in which PL and IL play opposing roles in the rodent threat response, the former driving threat expression, the latter threat extinction. Similarly, in human studies, the dACC and Brodmann 32-related mPFC areas appear to support threat responses and are hyperactive in PTSD associated with hyperarousal and threat emotional dysregulation. Furthermore, human data support subgenual and Brodmann 25-related mPFC areas (i.e., the vmPFC) in regulating/suppressing threat responses, supporting extinction of threat behaviors and fear emotions, and in providing top-down emotional control over amygdala and other subcortical regions.

However, there are other data to suggest that, more generally, PL encodes for the learning of rules, while IL allows for rule reversal [18]. Thus, the precise role for the mPFC is not yet entirely clear. The lens through which the “fear and threat” neuroscientists see these areas needs to become more aligned with how the “appetitive and addiction” and “cognitive control” neuroscientists view them. This will help obtain a more comprehensive overall perspective on these regions, aid in understanding their normal function, and provide better treatment approaches for a large number of disorders associated with their dysfunction. The integration of elegant and powerful circuit tools, from intersectional optogenetic and chemogenetic circuit dissection to cellular and genetically driven in vivo calcium imaging combined with behavior, are providing remarkable cell- and circuit-level appreciation of basic behavioral functions in rodents. In humans, new tools such as fMRI-guided TMS, as one example, are beginning to allow relatively rapid translation of circuit function to targeted, precision-medicine approaches for individually guided care.

Furthermore, as we have learned with subcortical structures, different cell types residing within the same brain structure may have dramatically different, sometimes opposing, functions. The mPFC has immense cellular heterogeneity, and we may expect, functional complexity. Future cell-type specific studies that dissect the circuit- and molecular-adaptations to threat learning within the mPFC will reveal additional layers of complexity to this system, and, hopefully, new therapeutic targets for PTSD. Tools, such as single cell RNA sequencing, are now being used across species from rodent to post-mortem human brain, allowing for molecular dissections to complement the above functional circuit dissections. Their translation across species will support both a scientific understanding of conservation of molecules and circuits across evolution for specific survival-related behaviors, as well as provide much more powerful targets for pharmacological and biological intervention using animal model systems targeting known conserved molecules and cell types for human therapeutic development.

In summary, while much remains to be learned, it is an extraordinarily exciting time for the field in which a great deal of convergence and replication has resulted in a fairly robust understanding of threat processing and its regulation by the PFC. How these areas encode and express threat memories over time is rapidly being dissected and integrating these data into our understanding of pathology in disorders such as PTSD is occurring. Integrating translational research in the coming years promises exciting new discoveries and approaches which may both greatly expand our knowledge of how the brain encodes behavior and also drive development of novel and robust new treatment approaches.
